# Protein transfer learning improves identification of heat shock protein families

**DOI:** 10.1371/journal.pone.0251865

**Published:** 2021-05-18

**Authors:** Seonwoo Min, HyunGi Kim, Byunghan Lee, Sungroh Yoon

**Affiliations:** 1 Department of Electrical and Computer Engineering, Seoul National University, Seoul, South Korea; 2 Department of Electronic and IT Media Engineering, Seoul National University of Science and Technology, Seoul, South Korea; 3 Department of Biological Sciences, Interdisciplinary Program in Bioinformatics, Interdisciplinary Program in Artificial Intelligence, ASRI, INMC, and Institute of Engineering Research, Seoul National University, Seoul, South Korea; Vellore Institute of Technology: VIT University, INDIA

## Abstract

Heat shock proteins (HSPs) play a pivotal role as molecular chaperones against unfavorable conditions. Although HSPs are of great importance, their computational identification remains a significant challenge. Previous studies have two major limitations. First, they relied heavily on amino acid composition features, which inevitably limited their prediction performance. Second, their prediction performance was overestimated because of the independent two-stage evaluations and train-test data redundancy. To overcome these limitations, we introduce two novel deep learning algorithms: (1) time-efficient DeepHSP and (2) high-performance DeeperHSP. We propose a convolutional neural network (CNN)-based DeepHSP that classifies both non-HSPs and six HSP families simultaneously. It outperforms state-of-the-art algorithms, despite taking 14–15 times less time for both training and inference. We further improve the performance of DeepHSP by taking advantage of protein transfer learning. While DeepHSP is trained on raw protein sequences, DeeperHSP is trained on top of pre-trained protein representations. Therefore, DeeperHSP remarkably outperforms state-of-the-art algorithms increasing F1 scores in both cross-validation and independent test experiments by 20% and 10%, respectively. We envision that the proposed algorithms can provide a proteome-wide prediction of HSPs and help in various downstream analyses for pathology and clinical research.

## Introduction

Heat shock proteins (HSPs) are stress-induced proteins that are highly conserved across organisms ranging from bacteria to humans [1]. HSPs participate in several cellular processes such as intercellular transportation and signal pathway modulation. Most importantly, HSPs play a pivotal role as molecular chaperones against unfavorable conditions, such as elevated temperature and inflammation [2]. They prevent irreversible aggregation of denatured proteins and assist protein folding for functional conformation. Because the dysfunction of HSPs may lead to fatal illness (*e.g.*, neurodegenerative disorders, cardiovascular diseases, and cancers), their identification has been an important problem in pathology and clinical research [3].

According to core functions and molecular weights [4], HSPs can be categorized into six major families: HSP20 (small HSPs), HSP40 (DnaJ proteins), HSP60 (GroEL proteins), HSP70 (DnaK proteins), HSP90 (HptG proteins), and HSP100 (Clp proteins). Traditional methods rely on nuclear magnetic resonance spectroscopy to identify HSP families [5]. However, as an exponential number of proteins are becoming available, time-consuming and resource-intensive processes of experimental annotation have become a serious disadvantage.

Therefore, numerous computational methods have been developed to identify HSP families (Table 1). They commonly used a support vector machine (SVM) classifier trained on a variety of sequence composition features, *e.g.*, pseudo amino acid composition (PAAC), dipeptide composition (DPC), and spaced-DPC (SDPC). While early methods [6, 7] only focused on classifying HSP sequences into one of the six HSP families, PredHSP [8] and ir-HSP [9] proposed two-stage algorithms to cope with non-HSP input sequences as well. They are based on sequence composition feature extraction, followed by two trained SVM classifiers. In the first stage, they used an SVM model to discriminate HSP sequences from non-HSP sequences. In the second stage, they used another SVM model to classify those predicted as HSPs into one of the six families. The main difference between the algorithms lies in the type of extracted features.

**Table 1:** Summary of related works.

Method	Feature	Model	Non-HSP	HSP Families
iHSP-PseRAAC [6]	PAAC	SVM	X	O
Ahmad *et al.* [7]	DPC	SVM	X	O
PredHSP [8]	DPC	SVM	O	O
ir-HSP [9]	SDPC	SVM	O	O

Previous studies have provided high-throughput methods for identifying HSP families. However, they had two major limitations. First, they relied heavily on the sequence composition features, focusing only on dipeptide statistics. Because they cannot capture more complex high-level information, it limited the performance of the previous algorithms. Second, their prediction performance was overestimated owing to biased experiments. During cross-validation, the second SVM was evaluated independently without considering the first SVM. This resulted in a higher number of true positives, although some of them already have been misclassified as non-HSPs in the first stage. Moreover, during additional tests, it was not ensured that the additional datasets were independent. We found that there are numerous sequences similar to those in the training dataset. The data redundancy also inevitably caused overrated evaluations.

With the advancement of deep learning, several studies have proposed deep learning models for bioinformatics [10]. As conventional machine learning models heavily rely on extracted features, machine learning researchers often focus on designing effective features for various tasks [11–13]. In contrast, deep learning models eliminate the laborious feature engineering and use deep neural networks to learn hierarchical representations from data. They showed that deep learning models, trained with a substantial amount of labeled data, can achieve state-of-the-art performance in various problems such as CRISPR activity and microRNA target prediction. [14, 15].

Transfer learning is an important cornerstone of deep learning. For example, in natural language processing, word representations are pre-trained using a huge amount of unlabeled text [16, 17]. The learned information can be transferred to a wide range of tasks by training task-specific models on top of the pre-trained word representations. The crux of transfer learning is how to pre-train representations. Several studies have proposed language model (LM)-based approaches that can exploit unlabeled data [16, 17]. Given a sentence, they train an LM such that a randomly masked word is predicted from representations of other words.

Similarly, a variety of studies have proposed LM-based approaches for protein transfer learning [18–22]. As evolutionary pressure constrains naturally occurring proteins to maintain indispensable functions, they could obtain implicit information underlying protein sequences even without any experimental annotations. Taking advantage of a large number of unlabeled protein sequences, it was demonstrated that pre-trained protein representations convey biochemical, structural, and evolutionary information. Therefore, pre-trained representations can help improve model performance in various protein biology tasks [23].

The key differences among the previous studies originate from two factors: (1) LM architecture and (2) the number of proteins used for pre-training (Table 2). In terms of the LM architecture, UniRep, PLUS-RNN, and SeqVec use recurrent neural networks (RNNs); ProtXLNet, ProtBERT, and ESM use transformers (TFMs). RNN-based models require less resources for both pre-training and producing representations. Although TFM-based models require significantly more resources, they are better at capturing long-term dependencies within proteins and can provide more informative representations [24]. The number of unlabeled proteins used in each study varied considerably. The LMs with more parameters were usually pre-trained with a larger number of proteins. Exceptionally, while ESM used the largest protein LM, it was pre-trained with a relatively small number of proteins. This can be attributed to its high-diversity dataset, which contains only representative proteins from clusters based on sequence identity [22].

**Table 2:** Pre-trained protein language models.

	Model	Parameter (M)	Dimensions	Proteins (M)
UniRep	RNN	18	1,900	24
PLUS-RNN	RNN	59	2,048	15
SeqVec	RNN	94	1,024	33
ProtXLNet	TFM	409	1,024	216
ProtBERT	TFM	421	1,024	2,122
ESM	TFM	669	1,280	27

In this work, we introduce two novel deep learning algorithms for the identification of HSP families. First, we propose time-efficient DeepHSP based on a convolutional neural network (CNN). It leverages (1) the representation learning capability of deep learning and (2) a one-stage algorithm trained to classify both non-HSPs and the six HSP families simultaneously. It outperforms state-of-the-art algorithms, despite taking 14–15 times less time for both training and inference. We further improve DeepHSP by taking advantage of protein transfer learning. We train the CNN model on top of pre-trained protein representations instead of the raw sequences used for DeepHSP. We denote the resulting model as DeeperHSP considering that the representations are obtained from a pre-trained deep neural network. We demonstrate that high-performance DeeperHSP remarkably outperforms state-of-the-art algorithms in both cross-validation and independent test experiments, increasing F1 score by 20% and 10%, respectively.

In summary, the contributions of our paper are as follows:

- We introduce time-efficient DeepHSP and high-performance DeeperHSP for the computational identification of HSP families.
- DeepHSP outperforms state-of-the-art algorithms, despite taking 14–15 times less time for both training and inference.
- Incorporating pre-trained protein representations and a CNN model, DeeperHSP remarkably outperforms state-of-the-art algorithms both in cross-validation and independent test experiments, increasing the F1 scores by 20% and 10%, respectively.
- All the data, codes, and pre-trained models are available at https://github.com/mswzeus/DeeperHSP.

## Materials and methods

### DeepHSP

**Fig 1:**
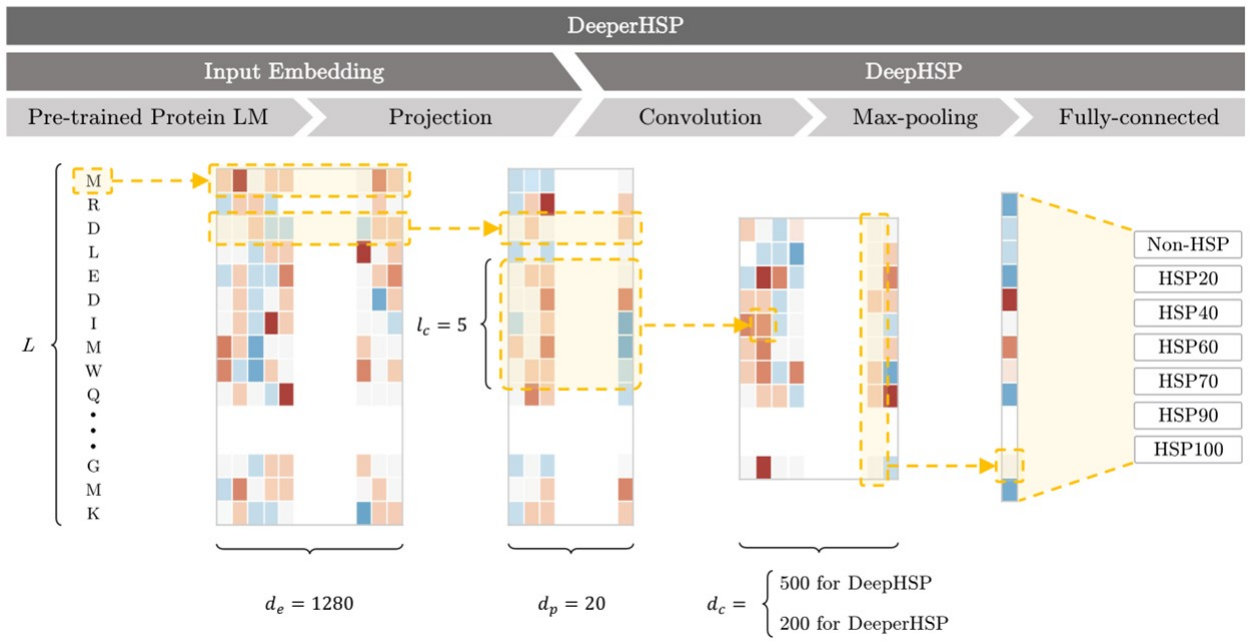
Overview of DeepHSP and DeeperHSP.

We propose time-efficient DeepHSP which categorizes a protein sequence into seven classes: non-HSP and the six major HSP families (Fig 1). Hereafter, we explain the CNN-based model step-by-step with an input protein sequence of variable-length *L* denoted as

$$\begin{array}{c} S=\left(s_{1},\ldots ,s_{L}\right),\;s_{i}\in \left\{\text{20}\;\text{standard}\;\text{amino}\;\text{acids}\right\}. \end{array}$$

Given a protein sequence *S*, DeepHSP first uses one-hot encoding to convert it into $X\in R^{L\times 20}$, a sequence of 20-dimensional vectors:

$$\begin{array}{c} X=\left\langle x_{1},\ldots ,x_{L}\right\rangle ,\;x_{i}=\text{OneHot}\left(s_{i}\right), \end{array}$$

such that all the elements in **x**_*i*_ are set to zero, except the element corresponding to *s*_*i*_, that is set to one.

Subsequently, the convolution and max-pooling layers compute hidden representations, $H\in R^{500}$, from the encoded input matrix:

$$\begin{array}{c} H=\text{MaxPool}(\text{Conv}(X)). \end{array}$$

The convolution layer uses *d*_*c*_ = 500 filters of length *l*_*c*_ = 5 followed by a rectified linear unit activation function. The filters can be regarded as position-weighted matrices similar to those used in traditional analyses [10]. They are convolved along protein sequences and trained to identify discriminative motifs. The global max-pooling layer computes the maximum value of the output of each filter. This helps us to obtain a fixed-length representation vector from the variable-length input sequence.

Finally, the fully-connected (FC) layer computes the outputs **P** from the representations:

$$\begin{array}{c} P=\text{FC}\left(H\right),\;P=\left(p_{1},\ldots ,p_{7}\right) \end{array}$$

where *p*_*c*_ denotes the probability of each class *c* of the input sequence, and $\sum_{c=1}^{7}p_{c}=1$. The FC layer uses dropout regularization and a softmax activation function. The former randomly zeroes some of the input vectors to help avoid overfitting. The latter normalizes the output vector so it can be interpreted as a probability distribution.

### DeeperHSP

The main limitation of DeepHSP originates from the one-hot encoding. It can only identify the amino acid in each position and cannot provide any other information. To tackle this problem, we propose high-performance DeeperHSP, which takes advantage of protein transfer learning. DeeperHSP embeds an input sequence using a pre-trained protein LM and a projection layer (Fig 1).

Given a protein sequence *S*, DeeperHSP uses a pre-trained protein LM to convert it into a sequence of *d*_*e*_-dimensional vectors, $E\in R^{L\times d_{e}}$:

$$\begin{array}{c} E=\left\langle e_{1},\ldots ,e_{L}\right\rangle ,\;E=\text{ProteinLM}\left(S\right). \end{array}$$

In contrast to one-hot encoding, which independently converts each amino acid, the protein LM computes representations as a function of the entire sequence. By leveraging a large number of unlabeled proteins through pre-training, they provide biochemical, structural, and evolutionary information that can help us to identify HSP families. Among a variety of pre-trained protein LMs, we used the largest ESM [22], which produces vectors of dimension *d*_*e*_ = 1280. The effects of different protein LMs are presented in the ablation studies.

The size of the representations, *d*_*e*_, is more than a couple of thousand dimensions. This may significantly increase the number of parameters in the following model. Therefore, DeeperHSP uses a projection layer to further embed **E** into vectors $Z\in R^{L\times d_{p}}$, where *d*_*p*_ = 20:

$$\begin{array}{c} Z=\left\langle z_{1},\ldots ,z_{L}\right\rangle ,\;z_{i}=\text{Proj}\left(e_{i}\right), \end{array}$$

where it independently embeds **e**_*i*_ into **z**_*i*_ with shared weights across different positions. The projection layer minimizes the additional number of parameters required for DeeperHSP.

Finally, DeeperHSP uses a CNN on top of the embedded input matrix. It utilizes the same CNN architecture as DeepHSP except that (1) its input *X* is replaced with *Z* and (2) its convolution layer uses *d*_*c*_ = 200 filters. The latter is to make the number of parameters of DeeperHSP (47K) similar to that of DeepHSP (54K). It helps us to clearly examine the effectiveness of the pre-trained representations used for DeeperHSP.

### Training of DeepHSP and DeeperHSP

For the training of both DeepHSP and DeeperHSP, we use class-weighted cross-entropy objective function defined as

$$\begin{array}{c} L=-\sum_{c=1}^{7}w_{c}\cdot y_{c}\text{log}(p_{c}),\\ fw_{c}=\sqrt{\frac{\text{max}(\text{number}\;\text{of}\;\text{samples}\;\text{in}\;\text{each}\;\text{class})}{\text{number}\;\text{of}\;\text{samples}\;\text{in}\;\text{class}\;c}}, \end{array}$$

where *y*_*c*_ ∈ {0, 1} denotes the label of each class for the input. Because training datasets are highly class-imbalanced, we use the class weights *w*_*c*_ to manually scale the training loss for each class.

We trained the models for 20 epochs using the Adam optimizer [25] with a mini-batch size of 100, a learning rate of 0.0004, and a dropout probability of 0.4. Note that for DeeperHSP, we left the pre-trained LM intact and only trained the projection layer and the CNN model. We used PyTorch [26] and Bio_Embeddings [27] libraries for model implementations and to obtain pre-trained representations, respectively.

## Results

### Datasets

#### Cross-validation dataset

For cross-validation experiments, we utilized the same dataset used in previous studies [8, 9]. Non-HSP sequences were randomly selected without homologous proteins from SwissProt [28]. HSP sequences were derived from HSPIR [4]. Thereafter, the proteins with ≥ 40% pairwise sequence similarity within the same family were removed using CD-HIT [29]. Finally, the non-HSP and HSP sequences containing non-standard amino acids were filtered out to obtain a cross-validation dataset (Table 3).

**Table 3:** Summary of cross-validation and independent test datasets.

Class	Cross-Validation Dataset	Independent Test Dataset
Non-HSP	9,965	500
HSP20	354	12
HSP40	1,257	52
HSP60	159	8
HSP70	278	53
HSP90	52	35
HSP100	81	20

#### Independent test dataset

Although previous studies used additional test datasets, they did not ensure that those were independent from the cross-validation dataset [8, 9]. Therefore, we curated a new independent test dataset to evaluate the generalization performance (Table 3). We randomly sampled non-HSP sequences from Pfam [30] and collected manually verified HSP sequences from three data sources, *i.e.*, HGNC [31], RICE [32, 33], and InterPro [34]. Most importantly, we used CD-HIT [29] to remove homogeneous proteins such that no two proteins from the cross-validation and test datasets have 40% or more pairwise sequence similarity within the same class. We filtered out about 80% of the 3,911 curated sequences and obtained an independent test dataset of 680 sequences.

### Feature extraction-based baselines

We compared the performance of DeepHSP and DeeperHSP with those of two state-of-the-art algorithms: PredHSP [8] and ir-HSP [9]. Because their codes are not publicly available, we re-implemented them using the Scikit-learn library [35]. First, we extracted DPC and SDPC features for PredHSP and ir-HSP, respectively. Then, we trained the radial basis function kernel SVM models. We selected SVM hyperparameters with the best performance among 144 configurations: 12 points of regularization penalty *C* and kernel coefficient *γ* that were evenly spaced between 10^3^ and 10^−3^.

To set competitive baselines, we made some modifications during the re-implementations. While PredHSP and ir-HSP are two-stage algorithms, we converted them into one-stage algorithms that classify both non-HSPs and the six HSP families simultaneously. We found that the latter performs better by utilizing all class information in a single integrated model. In addition, for ir-HSP, we removed random forest (RF)-based feature selection and used all 1600-dimensional SDPC features. We discovered that feature selection did not improve the classification performance. We report the performance of the modified baselines for the following cross-validation and independent results. The performance comparisons between the original and modified baselines are provided in the ablation studies.

### Cross-validation results

We evaluated the classification performance of PredHSP, ir-HSP, DeepHSP, and DeeperHSP using five-fold cross-validation. We used eight evaluation metrics: accuracy, F1 score, precision, recall, specificity, MCC, AUC-ROC, and AUC-PR. Because all the evaluation metrics, except for accuracy, are defined for binary classification, we used unweighted averages of the scores computed for each class.

First, we compared the overall classification performance (Table 4). The results show that the proposed DeepHSP and DeeperHSP significantly outperformed the state-of-the-art algorithms. The gap between ir-HSP and DeepHSP verifies the importance of deep learning. DeepHSP was able to learn discriminative representations that could not be captured using the sequence composition features. The performance improvement obtained by DeeperHSP demonstrates the effectiveness of the pre-trained protein representations. They provide a wealth of information learned from a large number of unlabeled protein sequences. By incorporating the pre-trained representations and the CNN model, DeeperHSP outperformed the previous algorithms in terms of all the evaluation metrics, notably increasing the F1 score by 20%.

**Table 4:** Comparison of overall classification performance using 5-fold cross-validation.

Model	Accuracy	F1 Score	Precision	Recall	Specificity	MCC	AUC-ROC	AUC-PR
PredHSP †	0.9128	0.6839	0.9044	0.5856	0.9409	0.6686	0.9496	0.7725
ir-HSP †	0.9483	0.8276	0.9437	0.7611	0.9678	0.8147	0.9725	0.8651
DeepHSP	0.9682	0.8613	0.9617	0.7984	0.9778	0.8554	0.9779	0.8931
DeeperHSP	**0.9927**	**0.9693**	**0.9745**	**0.9666**	**0.9957**	**0.9664**	**0.9947**	**0.9667**

^†^ Modified version for performance improvement.

Next, we compared their class-wise classification performance in terms of the F1 score (Table 5). The results show similar performance improvement trends. DeepHSP outperformed the previous algorithms for most classes. However, it did not perform well for the classification of HSP90, where the least number of training samples are available. This indicates the difficulty of training deep neural networks from scratch without sufficient data. In contrast, DeeperHSP provided the best classification performance for all classes. We can conclude that the pre-trained protein representations could help stabilize the training of the CNN model, particularly for classes with limited training data.

**Table 5:** Comparison of class-wise classification performance in terms of F1 score using 5-fold cross-validation.

Model	Non-HSP	HSP20	HSP40	HSP60	HSP70	HSP90	HSP100	Average
PredHSP †	0.9502	0.6353	0.7573	0.4181	0.6159	0.6558	0.7544	0.6839
ir-HSP †	0.9698	0.8190	0.8696	0.6175	0.8165	0.8315	0.8692	0.8276
DeepHSP	0.9812	0.8554	0.9540	0.7157	0.8149	0.8079	0.9001	0.8613
DeeperHSP	**0.9956**	**0.9873**	**0.9847**	**0.9607**	**0.9648**	**0.9152**	**0.9768**	**0.9693**

^†^ Modified version for performance improvement.

**Fig 2:**
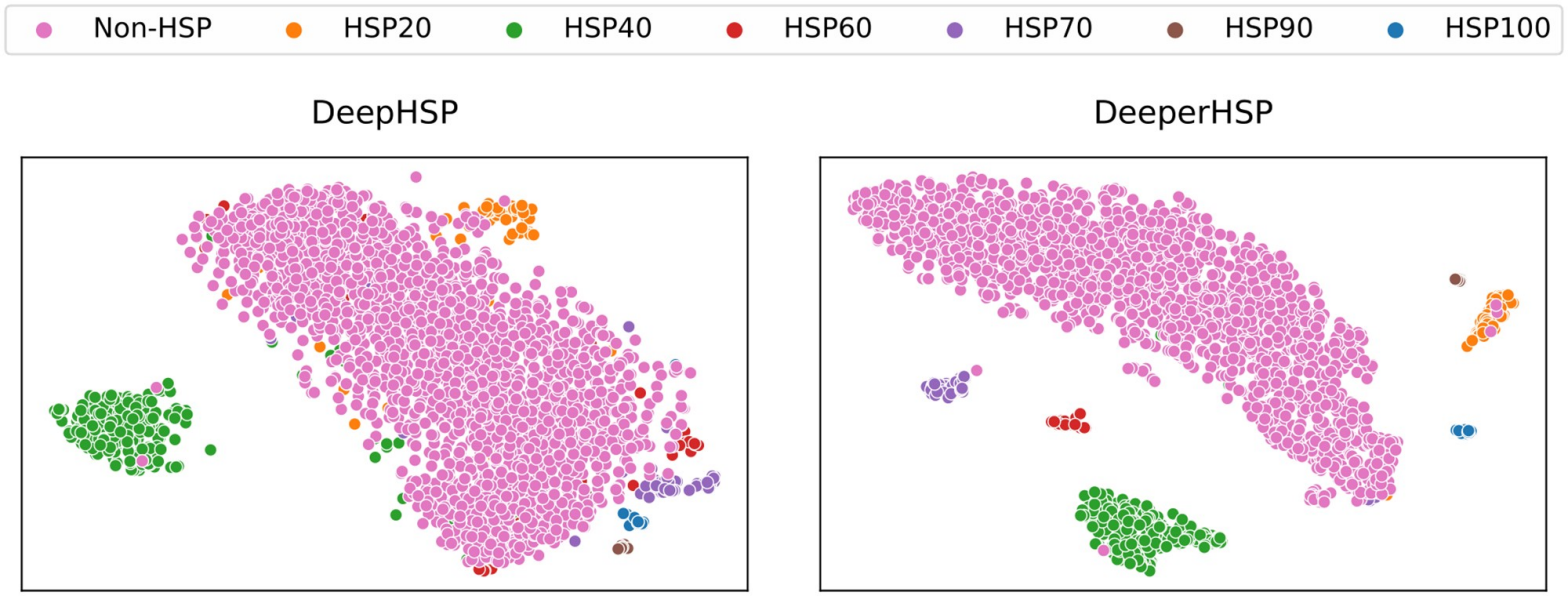
t-SNE plot of the latent representations of DeepHSP and DeeperHSP for the cross-validation dataset.

Finally, we examined the latent representations of DeepHSP and DeeperHSP. We used the t-distributed stochastic neighbor embedding (t-SNE) visualizations [36] with representations obtained from their penultimate layers (Fig 2). The latent representations of DeeperHSP are more clearly clustered into different groups according to their classes. By comparing the results, we confirmed the superiority of DeeperHSP over DeepHSP.

### Independent test results

We used an independent test dataset to evaluate the different algorithms. The results show that the proposed models consistently outperformed the previous algorithms (Table 6). In particular, compared to ir-HSP, DeeperHSP increased F1 score by 10%. Considering that DeeperHSP has less parameters than DeepHSP, it is remarkable that the pre-trained representations could improve the performance of the CNN model.

**Table 6:** Comparison of overall classification performance using independent test.

Model	Accuracy	F1 Score	Precision	Recall	Specificity	MCC	AUC-ROC	AUC-PR
PredHSP †	0.9324	0.8017	**0.9330**	0.7643	0.9698	0.7993	0.9515	0.8154
ir-HSP †	0.9456	0.8302	0.9005	0.8092	0.9780	0.8240	0.9677	0.8400
DeepHSP	0.9500	0.8340	0.8870	0.8303	0.9805	0.8292	0.9431	0.8079
DeeperHSP	**0.9691**	**0.9126**	0.9230	**0.9077**	**0.9902**	**0.9043**	**0.9692**	**0.8747**

^†^ Modified version for performance improvement.

**Fig 3:**
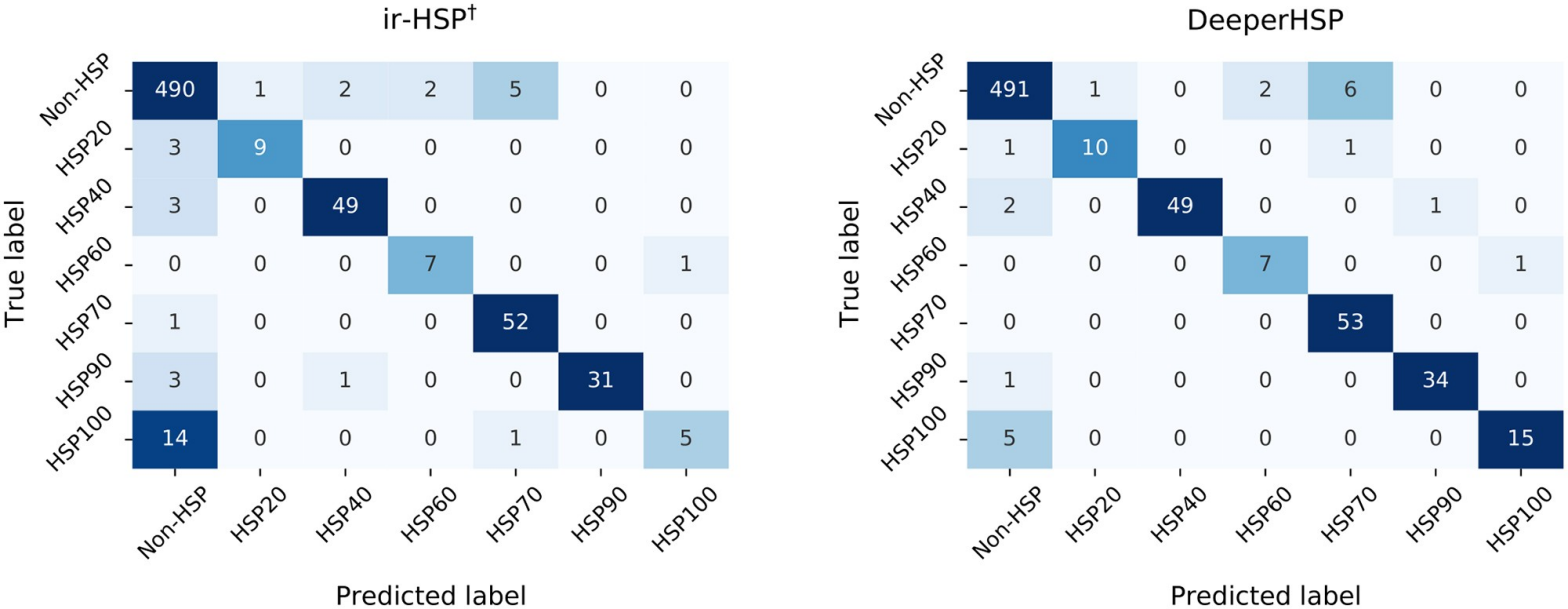
Confusion matrices of the modified ir-HSP and DeeperHSP predictions for the independent test dataset.

We also present the confusion matrices of the ir-HSP and DeeperHSP predictions for the independent test dataset (Fig 3). We can observe that DeeperHSP provides better classification performance. It correctly classified the majority of the HSP100 samples, where ir-HSP did not perform satisfactorily. Meanwhile, the confusion matrices of ir-HSP and DeeperHSP exhibited similar misclassification patterns. In particular, among the 21 samples misclassified by DeeperHSP, 18 samples were also misclassified into the same incorrect classes by ir-HSP. This might imply that there are limitations to sequence-based identification of HSP families and additional structural information is required for performance improvement.

### Running time

We compared the running time required for each algorithm. We report training time for the cross-validation dataset and inference time for the independent test dataset. Note that we used single CPU for the SVM-based algorithms and single GPU for the deep learning-based algorithms.

The results show that the proposed models have trade-off between performance and time-efficiency (Table 7). DeepHSP showed small improvement in performance but a strong advantage in time-efficiency. It was about 14–15 times faster than ir-HSP for both training and inference. On the other hand, while DeeperHSP demonstrated the best performance, it exhibited the longest running time. This was largely due to the time required for obtaining pre-trained representations. Based on these observations, we believe that the different strengths of DeepHSP and DeeperHSP can provide different options based on users’ demands.

**Table 7:** Comparison of running time required for each algorithm.

Model	Training (seconds)	Inference (seconds)
PredHSP †	315	5
ir-HSP †	1,265	14
DeepHSP	80	1
DeeperHSP	2,112	120

^†^ Modified version for performance improvement.

### Ablation studies

#### Feature extraction-based baselines

For competitive baselines, we explored both one- and two-stage algorithms based on different combinations of features and classifiers. We considered six types of features [37]: amino acid composition (AAC), DPC, SDPC, PAAC, composition transition distribution (CTD), and Moreau-Broto auto-correlation (MBAuto). We also considered six types of classifiers [35]: XGBoost, RF, Lasso, Ridge, ElasticNet, and SVM. For each classifier, we selected its hyperparameters with the best performance among 144 configurations.

**Fig 4:**
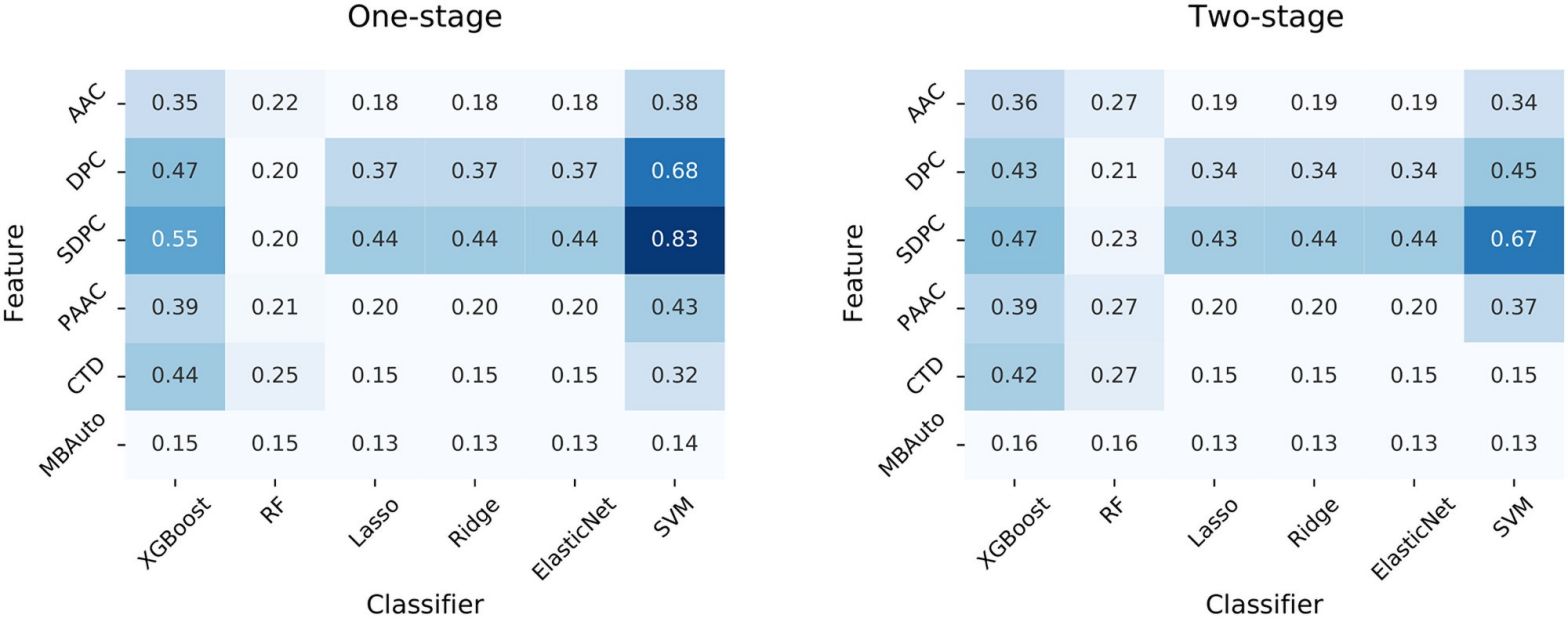
Heatmaps of the F1 scores obtained from baseline algorithms using 5-fold cross-validation. One-stage algorithms classify both non-HSPs and the six HSP families simultaneously. Two-stage algorithms use two models to filter out non-HSPs and classify the remaining HSPs into the six families.

Fig 4 presents heatmaps of the F1 scores obtained from the different algorithms using five-fold cross-validation. We can observe that the one-stage algorithms performed better than the two-stage algorithms. Comparing the different combinations of features and classifiers, two of them clearly stand out. These are the modified versions of PredHSP and ir-HSP, which are based on SVM classifiers trained using DPC and SDPC features, respectively.

We further examined whether techniques used in previous works could improve the classification performance [9, 38]. We used a one-stage SVM model trained on the SDPC features as a baseline model. Then, we additionally adopted either (1) RF-based feature selection to choose a smaller number of important features or (2) the syntactic minority oversampling technique (SMOTE) [39] to deal with class imbalance. We discovered that both techniques led to lower F1 scores of 0.71 and 0.63, respectively.

#### Pre-trained protein representations

We explored various pre-trained protein LMs to obtain representations for DeeperHSP: UniRep, SeqVec, PLUS-RNN, ProtXLNet, ProtBERT, and ESM.

**Fig 5:**
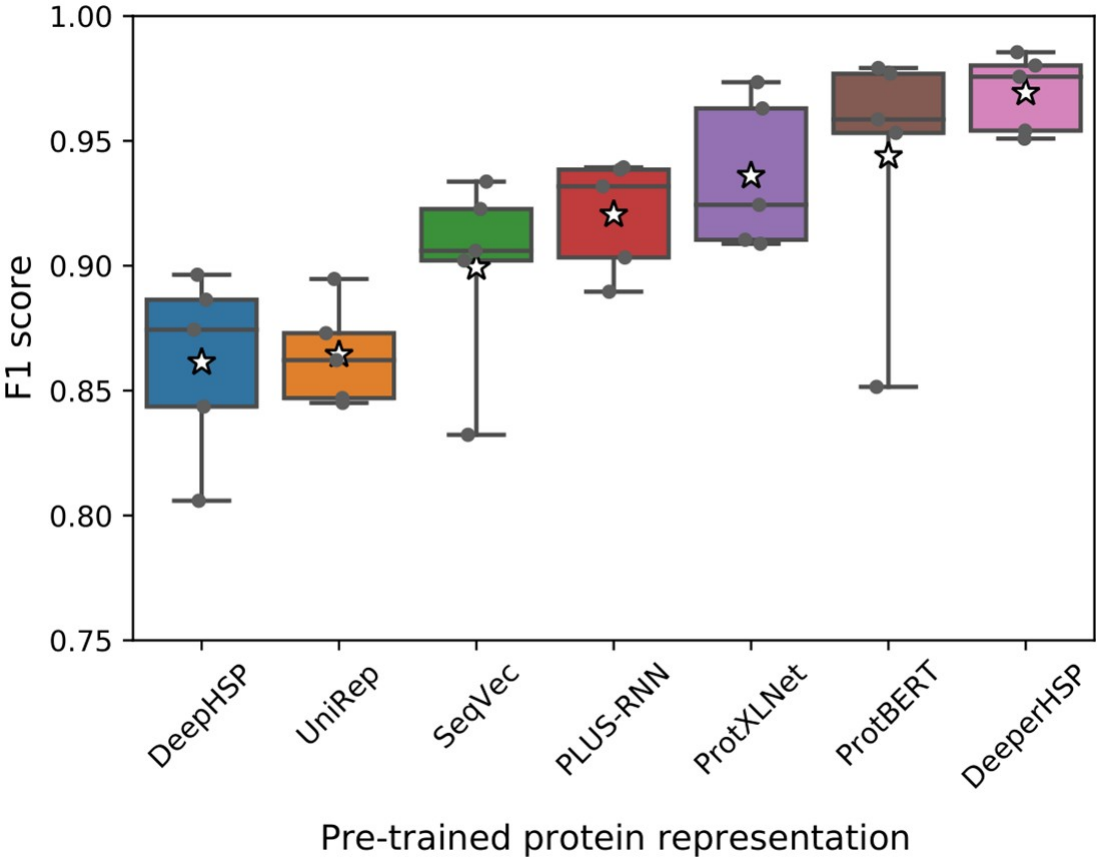
Boxplot of the F1 scores obtained from DeeperHSP with different pre-trained protein representations using 5-fold cross-validation.

We compared the performance of DeeperHSP with different LMs using five-fold cross-validation (Fig 5). Additionally, as a baseline, we include the performance of DeepHSP in the leftmost column. Each box denotes the quartiles of F1 scores, and the star denotes their average. The boxplot shows that all LMs improve the average classification performance compared to DeepHSP. Taking advantage of a large number of unlabeled proteins, the pre-trained protein representations provide a wealth of information that cannot be learned from one-hot encoding.

While all the pre-trained protein LMs help in the identification of HSP families, their level of performance improvement varies significantly. The small gap between OneHot (*i.e.*, DeepHSP) and UniRep indicates that a sufficient number of parameters are required to obtain a moderate increase (Table 2). LMs with more parameters generally provide more performance improvement. For example, the larger TFM-based LMs outperformed the RNN-based LMs, and the largest ESM showed the best performance. One exception is that although PLUS-RNN has fewer parameters than SeqVec, it exhibits better performance. We conjecture that this can be attributed to its additional protein-specific pre-training objective, which can better capture structural information of protein sequences than those solely pre-trained with an LM [19].

## Conclusion

In this paper, we proposed two novel deep learning algorithms that classify both non-HSPs and the six HSP families simultaneously. The time-efficient DeepHSP uses a CNN model that identifies the HSP families faster and more accurately than the alternatives. Furthermore, the high-performance DeeperHSP leverages protein transfer learning to improve performance. It trains the CNN model on top of the pre-trained protein representations instead of the one-hot encoded protein sequences. Our experimental results showed that DeeperHSP remarkably outperformed the state-of-the-art algorithms. It increased F1 scores by 20% and 10% on the cross-validation and independent test datasets, respectively. We envision that the proposed algorithms can provide a proteome-wide prediction of HSPs and help various downstream analyses for pathology and clinical research.

Although the proposed algorithms have a clear advantage over the previous approaches, there are still some limitations and room for further improvement. First, there are trade-offs between the running time and classification performance. We expect that a lightweight LM would be able to greatly reduce the running time for obtaining pre-trained protein representations [40]. This will enable the development of both time-efficient and high-performance algorithms for the identification of HSP families. Second, they only focused on classifying non-HSPs and HSP families. It would be valuable to develop a more comprehensive model that can provide additional information on other protein types and functions [41]. Finally, we believe it would also be interesting to extend this work to recent research topics in machine learning such as interpretability [42, 43] and security [44–47].
